# Clinical and Microbiological Effects of Smoking on Lithium Disilicate Endocrowns: An Age-Stratified Cross-Sectional Study

**DOI:** 10.3390/dj14010015

**Published:** 2026-01-01

**Authors:** Gabriela Popa, Dorin Ioan Cocoș, Gabriel Valeriu Popa, Andrei Iliescu, Cristina-Mihaela Popescu, Ada Stefanescu

**Affiliations:** 1 Faculty of Medicine and Pharmacy, Medical-Pharmaceutical Research Center “Dunărea de Jos” University, 800008 Galati, Romania; gabriela.popa@ugal.ro (G.P.); cristina.popescu@ugal.ro (C.-M.P.); ada.stefanescu@ugal.ro (A.S.); 2 Research Center of Dental Medicine, “Carol Davila” University of Medicine and Pharmacy, 020022 Bucharest, Romania; prof.andrei.iliescu@gmail.com; 3 Dental-Medicine Department, Faculty of Medicine and Pharmacy, “Dunărea de Jos” University of Galati, 800201 Galati, Romania; 4 “Sf. Ioan” Clinical Emergency Pediatric Hospital in Galati, 800487 Galati, Romania

**Keywords:** dentistry, lithium disilicate endocrowns, oral microbiome, periodontal pathogens, plaque accumulation, posterior teeth, prosthodontics, salivary pH, smoking

## Abstract

**Background:** Smoking alters oral ecological balance, yet its influence on posterior teeth restored with lithium disilicate endocrowns is insufficiently documented. This study assessed the clinical and microbiological impact of smoking on the peri-coronal environment of endocrown-restored teeth, using an age-stratified approach to evaluate cumulative effects. **Methods:** A cross-sectional study was conducted on 100 adults, equally divided into smokers and non-smokers. Salivary pH, papillary bleeding index, and plaque index were clinically recorded. Subgingival samples collected from endocrown-restored posterior teeth were analyzed using a polymerase chain reaction (PCR) assay targeting major periodontal pathogens. Age-related variation in clinical and microbiological parameters was examined using one-way analysis of variance (ANOVA), followed by Tukey’s HSD post hoc test. **Results:** Smokers showed consistently lower salivary pH and higher plaque accumulation across all age groups. Gingival bleeding was reduced in younger smokers but increased in older individuals. Microbiological analysis identified markedly elevated levels of orange-complex organisms in smokers, including *Prevotella intermedia* and *Fusobacterium nucleatum*. Clinically, endocrowns in smokers presented more frequent marginal degradation, localized inflammation, and early signs of recurrent caries. These effects intensified with age. **Conclusions:** Smoking adversely modifies the peri-coronal biological environment of lithium disilicate endocrowns by increasing acidity, promoting plaque maturation, and supporting dysbiotic microbial communities. Age further amplifies these changes. Considering smoking status and patient age during treatment planning may improve long-term restorative outcomes.

## 1. Introduction

Dental caries and periodontal disease remain the most prevalent oral pathologies worldwide, driven by biofilm maturation, acidic shifts in the oral environment, and host–microbial interactions [1,2,3]. Gingival inflammation represents the earliest clinical manifestation of biofilm accumulation, marked by elevated inflammatory mediators and reversible soft-tissue changes [4,5]. Persistent dysbiosis favors progression toward periodontitis, facilitated by complex microbial communities embedded in an extracellular matrix that enhances adhesion, metabolic cooperation, and resistance to antimicrobial agents [6,7,8,9].

Among behavioral determinants, tobacco smoking induces measurable changes in salivary biochemistry, microbial composition, and periodontal tissue response. Smokers exhibit lower salivary pH, increased plaque accumulation, and higher levels of pathogenic species, such as *Aggregatibacter actinomycetemcomitans*, *Porphyromonas gingivalis*, and *Fusobacterium* spp., which are strongly associated with advanced periodontal breakdown [10,11,12]. Tobacco exposure disrupts host immunity through vasoconstriction, altered cytokine profiles, and reduced gingival bleeding response, masking early signs of inflammation while promoting deeper destructive processes. At the microbial level, leukotoxin-producing strains of *A. actinomycetemcomitans* intensify epithelial injury and inflammatory signaling, including Poly(ADP-Ribose) Polymerase 1 (PARP-1) overexpression [13,14].

Within mature periodontal biofilms, orange-complex microorganisms, particularly *Fusobacterium nucleatum*, play a central role in biofilm maturation by acting as ecological bridges between early and late colonizers. This bridging function facilitates interspecies interactions, bacterial adhesion, and the transition toward more pathogenic microbial communities. Moreover, different *Fusobacterium* subspecies have been shown to differentially influence biofilm architecture and community organization [5,15,16]. In this context, preventive strategies centered on individualized oral hygiene instruction can attenuate biofilm accumulation and improve clinical outcomes even in high-risk groups [17].

Beyond periodontal and salivary alterations, smoking also affects the structural integrity of posterior teeth, thereby influencing restorative treatment choices. Endocrown restorations depend on adhesive retention, the presence of adequate supra-gingival coronal structure generally at least 2 mm circumferentially, and the availability of a stable pulp chamber for macromechanical anchorage [18,19,20]. Posterior teeth exposed to chronic acidity, increased plaque load, and heightened caries susceptibility may experience more extensive structural loss, potentially reducing their suitability for conservative adhesive restorations such as endocrowns [21,22]. Variations in salivary pH, plaque accumulation, and microbial burden between smokers and non-smokers may therefore influence both the initial clinical condition and the long-term prognosis of endocrown-restored teeth [18,20].

This study aimed to assess the effect of smoking and age on salivary pH, plaque accumulation, gingival bleeding, and subgingival microbial composition around lithium disilicate endocrown-restored posterior teeth by comparing smokers and non-smokers across different age groups.

## 2. Materials and Methods

### 2.1. Study Design and Ethical Approval

This observational, cross-sectional study included 100 adult patients who had previously received endocrown restorations on at least one posterior tooth. Participants were divided equally into two groups, non-smokers (*n* = 50) and active smokers (*n* = 50), each smoker reporting a minimum history of four consecutive years of tobacco use. A minimum smoking history of four years was required to ensure chronic tobacco exposure sufficient to induce stable salivary, microbiological, and periodontal alterations, rather than transient or reversible changes associated with short-term smoking. Epidemiological evidence supports that prolonged tobacco use is significantly associated with persistent periodontal alterations and microbial dysbiosis, with risk and severity increasing with longer duration of exposure [23]. For age-stratified analysis, participants were further categorized into seven predefined age groups: 19–25, 26–35, 36–45, 46–55, 56–65, 66–75, and ≥76 years. All patients were enrolled between June and August 2024 from a private dental clinic in Brăila, Romania. The study followed the principles of the Declaration of Helsinki and received approval from the Ethics Committee of “Dunărea de Jos” University of Galați (CEU/no RF2908/17.06.2024). Written informed consent was obtained from all participants.

The primary endpoint of the study was salivary pH measured adjacent to endocrown-restored posterior teeth. Sample size estimation was based on effect sizes reported in previous clinical studies comparing salivary pH and plaque-related parameters between smokers and non-smokers, indicating that a minimum of 40–45 participants per group is sufficient to detect moderate between-group differences (α = 0.05, power = 80%) [10,15,24]. To ensure adequate statistical robustness, account for age stratification, and compensate for potential biological variability, 50 participants were included in each group.

### 2.2. Inclusion and Exclusion Criteria

Inclusion criteria were age ≥ 18 years, presence of at least one molar or premolar restored with an endocrown, absence of antibiotic or anti-inflammatory medication in the past three months, and willingness to provide biological samples and clinical data. Exclusion criteria included pregnancy or lactation, uncontrolled systemic diseases (such as diabetes or autoimmune disorders), periodontal or orthodontic treatment within the previous six months, and use of antiseptic mouth rinses within one month before the examination.

### 2.3. Clinical Examination

Clinical evaluation included the assessment of salivary pH, papillary bleeding index (PBI), dental plaque index (PI), and the structural condition of posterior teeth restored with endocrowns. All examinations were performed by a calibrated clinician under standardized clinical conditions. Observer calibration was performed before the study through repeated clinical assessments on a pilot group of patients not included in the final analysis. Intra-examiner reliability for the papillary bleeding index and plaque index was evaluated using repeated measurements, yielding consistent agreement across assessments.

Salivary pH: Resting saliva was collected in sterile containers after participants refrained from smoking, eating, drinking, and toothbrushing for at least one hour. Measurements were obtained using the Saliva-Check Buffer test (GC Corp., Tokyo, Japan) and interpreted according to the standard colorimetric scale, following previously published protocols [25].

Papillary Bleeding Index (PBI) was recorded using conventional periodontal probing and expressed as mean values per patient, following established clinical protocols for gingival bleeding assessment [26]. Dental plaque accumulation was assessed using the Plaque Index described by Silness and Löe, with scores expressed as mean values per patient [27,28].

Assessment of Endocrown-Restored Teeth: All included molars and premolars restored with endocrowns were examined for marginal integrity, supra-gingival structural continuity, presence of secondary caries, restoration stability, and periodontal tissue response. Clinical findings were categorized as intact restoration, marginal degradation, recurrent caries, endodontic complications, or signs of structural compromise.

All included posterior teeth were previously restored with Computer-aided design (CAD) and Computer-aided manufacturing (CAM) endocrowns fabricated from lithium disilicate ceramic (IPS e.max CAD, Ivoclar Vivadent AG, Schaan, Liechtenstein). All teeth presented at least 2 mm of circumferential supra-gingival enamel, fulfilling standard eligibility criteria for adhesive endocrown retention. The restorations were adhesively luted using a dual-cure resin cement (Variolink Esthetic DC, Ivoclar Vivadent AG, Schaan, Liechtenstein), according to the manufacturer’s recommendations. Only clinically stable endocrowns with intact occlusion and no history of debonding or fracture were included in the analysis. Only one endocrown-restored posterior tooth per patient was included in the analysis to avoid clustering effects and ensure independence of observations. When multiple endocrown restorations were present, a single clinically eligible restoration was selected according to predefined inclusion criteria.

### 2.4. Microbiological Analysis

Subgingival biofilm samples were collected from the gingival sulcus adjacent to endocrown-restored teeth using sterile absorbent paper points (ISO size 30, Dentsply Sirona, Charlotte, NC, USA), kept in situ for 10–20 s after isolation and gentle drying of the area, following standardized periodontal microbiological sampling procedures [16]. Samples were transported under controlled temperature to MIP Pharma GmbH (Blieskastel, Germany) for quantitative real-time PCR analysis using the PET Plus^®^ diagnostic panel, in accordance with established real-time PCR methodologies for periodontal diagnostics [29]. Quantitative real-time PCR analysis was performed using a commercially available real-time PCR system at MIP Pharma GmbH (Blieskastel, Germany). The targeted periodontal pathogens included *Aggregatibacter actinomycetemcomitans*, *Prevotella intermedia*, *Prevotella melaninogenica*, *Fusobacterium nucleatum*, and *Eubacterium nodatum*.

### 2.5. Statistical Analysis

Statistical analysis was performed using IBM SPSS Statistics version 28.0 (IBM Corp., Armonk, NY, USA). Before inferential analysis, data distribution was assessed for normality using the Shapiro–Wilk test, complemented by visual inspection of histograms and Q–Q plots. As normality assumptions were met, parametric analyses were conducted using one-way ANOVA, with *p* < 0.05 considered statistically significant. Continuous variables were expressed as mean ± standard deviation (SD). Post hoc comparisons were performed using Tukey’s HSD test. Additional analyses evaluated the relationships between smoking status, microbial load, salivary pH, and the clinical condition of endocrown restorations.

## 3. Results

The study population comprised 100 adult patients, equally divided into smokers (n = 50) and non-smokers (n = 50), distributed across seven predefined age groups (19–25, 26–35, 36–45, 46–55, 56–65, 66–75, and ≥76 years), with all participants presenting at least one posterior tooth restored with a lithium disilicate endocrown.

Clinical and microbiological parameters were evaluated in patients presenting with posterior teeth restored with endocrowns, divided into smokers and non-smokers. The analysis focused on how tobacco exposure and age influenced salivary pH, plaque accumulation, gingival inflammation, microbial load, and the clinical condition of endocrown restorations.

Table 1 summarizes the salivary pH, papillary bleeding index, and dental plaque index recorded in the vicinity of posterior teeth restored with endocrowns in both groups.

Data are presented as mean ± SD. Differences between smokers and non-smokers across age strata were assessed using one-way ANOVA. Salivary pH: *p* < 0.001; Papillary Bleeding Index: *p* < 0.001; Plaque Index: *p* < 0.001.

Smokers exhibited consistently lower salivary pH values (6.40 ± 0.25) and higher plaque accumulation (2.27 ± 0.36) compared with non-smokers (6.77 ± 0.21 and 0.76 ± 0.24, respectively), with these differences reaching statistical significance (*p* < 0.001 for all parameters; Table 1). These differences became more pronounced among participants aged 46 or older, who showed steeper increases in plaque deposition and gingival inflammation. The overall trend indicates that smoking is associated with a less favorable peri-coronal environment around endocrown restorations, which may influence the stability of surrounding tissues and the long-term behavior of adhesive ceramic restorations.

Following the clinical assessment, microbiological analysis was performed on subgingival biofilm samples collected adjacent to endocrown-restored posterior teeth. The corrected values presented in Table 2 indicate consistent quantitative differences between smokers and non-smokers in both total microbial load and the distribution of orange-complex pathogens. Smokers exhibited significantly higher overall bacterial counts (*p* = 0.04), with markedly elevated levels of *Prevotella intermedia* (*p* < 0.001) and *Fusobacterium nucleatum* (*p* < 0.001), reflecting a shift toward a more anaerobic and dysbiotic microbial profile (Table 2). This microbial pattern suggests that smoking creates a biologically less favorable peri-coronal environment, characterized by enhanced plaque maturation and greater inflammatory potential around the margins of lithium-disilicate endocrowns. Such conditions may predispose smokers to marginal instability and an increased risk of secondary caries at the adhesive interface. Table 2 summarizes the mean values, standard deviations, and corresponding *p*-values, confirming that the differences observed between the two groups are statistically significant across all key microbial parameters.

**Figure 1:**
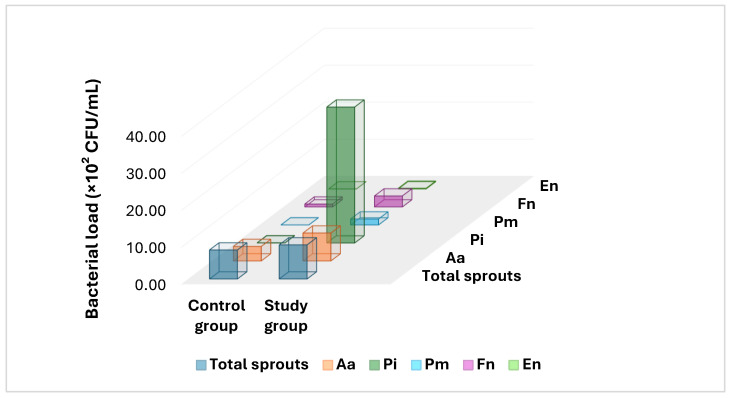
Quantitative distribution of orange complex and associated pathogens collected from the sulcus adjacent to endocrown-restored posterior teeth in control and study groups. (Aa, *Aggregatibacter actinomycetemcomitans*; Pi, *Prevotella intermedia*; Pm, *Prevotella melaninogenica*; Fn, *Fusobacterium nucleatum*; En, *Eubacterium nodatum*).

To illustrate the microbial differences around endocrown-restored posterior teeth, a graphical representation of the quantitative distribution of orange complex pathogens and associated species was generated. As shown in Figure 1, smokers exhibited a markedly higher microbial load adjacent to endocrown restorations, particularly for *Prevotella intermedia* and *Fusobacterium nucleatum* (orange complex), compared with non-smokers. Figure 1 presents the quantitative distribution of total microbial load and selected orange complex periodontal pathogens, expressed as mean values (×10^2^ CFU/mL) for *Aggregatibacter actinomycetemcomitans* (Aa), *P. intermedia* (Pi), *P. melaninogenica* (Pm), *Fusobacterium nucleatum* (Fn), and *Eubacterium nodatum* (En). The substantial elevation of Aa, Pi, and Fn in smokers indicates a pronounced microbial shift associated with tobacco exposure in the peri-coronal environment of endocrown restorations, which may influence their long-term clinical performance.

To evaluate the influence of age on clinical parameters measured around endocrown-restored posterior teeth, a one-way ANOVA was performed on the dental plaque index, papillary bleeding index, and salivary pH. Table 3 summarizes the results and shows statistically significant differences across age groups for all three variables. The analysis revealed that age had a significant effect on plaque accumulation (F = 3.58, *p* = 0.006), gingival bleeding (F = 12.44, *p* = 0.001), and salivary pH (F = 5.70, *p* = 0.001). These findings indicate a progressive age-related deterioration of the peri-coronal environment surrounding endocrown restorations, more evident in older individuals.

A separate one-way ANOVA was performed within the smoker group alone to determine whether age remained a significant influencing factor for the clinical parameters measured around endocrown-restored posterior teeth. As shown in Table 4, age continued to exert a statistically significant effect on all three variables. Plaque index (F = 4.23, *p* = 0.002), papillary bleeding index (F = 5.27, *p* < 0.001), and salivary pH (F = 5.17, *p* < 0.001) all demonstrated age-dependent variation. These findings indicate that, even when analyzed independently, smokers experience progressive deterioration of the peri-coronal environment with increasing age, reinforcing the cumulative impact of tobacco exposure and aging on endocrown-restored teeth.

### Clinical Condition of Endocrown Restorations

All evaluated endocrowns were fabricated from lithium disilicate (IPS e.max CAD). Clinical assessment revealed clear differences between smokers and non-smokers in the peri-coronal environment of these restorations. Non-smokers generally showed intact marginal adaptation, stable supra-gingival contours, and minimal soft-tissue inflammation. In smokers, superficial marginal degradation and localized discoloration at the adhesive interface were observed more frequently, together with a higher prevalence of gingival inflammation around the ceramic margins. These findings paralleled the increased plaque accumulation, lower salivary pH, and higher orange-complex bacterial load recorded in the smoking group, indicating a less favorable clinical and microbial environment for the long-term stability of lithium disilicate endocrowns. Overall, these findings indicate that smoking is associated with a less favorable peri-coronal environment for endocrown restorations, with potential implications for long-term restorative stability, periodontal health, and the reliability of adhesive interfaces.

## 4. Discussion

This study evaluated the clinical and microbiological effects of smoking on posterior teeth restored with endocrowns, using an age-stratified approach to capture both early and cumulative changes. The results show that smoking is associated with altered salivary conditions, increased plaque accumulation, modified gingival responses, and a higher prevalence of periodontal pathogens, supporting previous observations that tobacco promotes dysbiosis and accelerates periodontal deterioration [10,30].

Smokers consistently showed lower salivary pH across all age categories, with greater acidity in older adults. This trend is consistent with reports linking tobacco exposure to reduced salivary buffering capacity due to thermal injury and chemical components that affect glandular function [6]. Acidic saliva favors acidogenic and aciduric species, facilitating enamel demineralization and biofilm maturation [1,5]. Such changes are relevant for adhesive restorative procedures because they compromise the stability of enamel margins, which are essential for successful endocrown retention [18].

Plaque accumulation was significantly higher in smokers, particularly after mid-adulthood. Tobacco promotes biofilm maturation by lowering oxygen tension, shifting redox potential, and creating nutrient profiles favorable to proteolytic Gram-negative species [8,10]. This microbial pattern was reflected clinically: younger smokers exhibited reduced bleeding, likely due to nicotine-induced vasoconstriction, whereas older smokers showed increasing gingival inflammation. This dual pattern, masked early inflammation followed by rapid progression, is consistent with the documented vascular and immunologic effects of nicotine [4,9,11].

Microbiological analysis demonstrated a notable increase in orange complex pathogens among smokers, especially *Prevotella intermedia* and *Fusobacterium nucleatum*. Higher levels of *Aggregatibacter actinomycetemcomitans* observed in smokers also reflect its increased virulence under tobacco exposure, including enhanced leukotoxin activity and activation of host inflammatory pathways such as PARP-1 expression [13].

Clinically, these microbial and biochemical alterations were reflected in the condition of endocrown restorations. Non-smokers exhibited good marginal integrity and minimal inflammation, whereas smokers showed more frequent marginal degradation, localized discoloration, and early signs suggestive of recurrent caries. These findings match the observed shifts in pH, plaque accumulation, and pathogen load. Age amplified these effects, with older smokers presenting the most unfavorable peri-coronal conditions. These trends are consistent with the cumulative impact of aging, reduced salivary protection, and increasing microbial complexity [3,12,31].

An additional material-related consideration involves the adhesive behavior of lithium disilicate ceramics. IPS e.max endocrowns rely on a well-mineralized enamel substrate for predictable micromechanical and chemical adhesion [32]. A chronically acidic oral environment, common in smokers, can accelerate hydrolytic degradation of the resin–enamel interface, reduce bond durability, and facilitate early marginal breakdown. Moreover, decreased pH promotes superficial enamel demineralization, limiting the amount of sound cervical structure available for bonding and compromising long-term restorative stability. These mechanisms emphasize the sensitivity of adhesive ceramic systems to salivary acidity and reinforce the clinical significance of smoking-associated peri-coronal changes [32,33].

Lithium disilicate is highly dependent on a stable adhesive interface and a healthy peri-coronal environment to ensure predictable long-term performance [18,19,20]. The present findings indicate that smokers develop a more acidic, plaque-retentive, and bacterially enriched microenvironment [1,5,10,15,16], conditions that may compromise marginal stability and increase the risk of recurrent caries around endocrown restorations. These observations align with previous reports describing the sensitivity of lithium disilicate endocrowns to adhesive degradation and marginal biofilm accumulation, particularly under conditions of altered salivary pH and elevated levels of anaerobic pathogens [18,19,20].

The results highlight the importance of tailored periodontal and preventive strategies for smokers. Regenerative approaches such as advanced platelet-rich fibrin +(A-PRF+) have shown potential benefits in nicotine-compromised tissues by improving local healing capacity [34]. Additionally, personalized oral hygiene interventions have demonstrated measurable improvements in plaque control and inflammation, even in high-risk groups [17]. These strategies may help mitigate the unfavorable conditions identified in the present study.

From a restorative perspective, the findings are clinically relevant. Endocrown success relies on stable enamel margins, a low-inflammation environment, and predictable adhesion [18,19,20]. The acidic, plaque-rich, and microbially imbalanced environment observed in smokers may compromise these requirements, potentially affecting long-term performance. Smoking status and patient age should therefore be considered important prognostic factors when planning endocrown therapy. These findings may influence clinical decision-making, including the selection of restorative materials, the frequency of professional prophylaxis, and the establishment of shorter recall intervals for smokers.

Despite the cross-sectional design, the observed clinical and microbiological differences suggest patterns that warrant confirmation through prospective studies. Longitudinal investigations spanning 2–5 years would allow direct assessment of how smoking-driven changes in salivary pH, plaque maturation, and microbial dysbiosis influence the long-term survival, marginal stability, and adhesive performance of lithium disilicate endocrowns. Such studies could also clarify temporal interactions between aging, smoking intensity, and restorative degradation, offering stronger evidence for individualized follow-up protocols. Moreover, it could be interesting to test Microbiological Effects of Smoking on Endocrowns in combination with recently introduced adjunctive treatments that can alter oral parameters in periodontal patients, such as Ozone [35], and probiotics [36], to understand their mutual effect on periodontal tissues and prosthodontic frameworks.

### Strengths and Limitations

This study offers several methodological and analytical strengths. The age-stratified design provides a detailed perspective on how smoking affects the peri-coronal environment of endocrown-restored posterior teeth at different stages of adulthood, an aspect rarely explored in restorative or periodontal research. The combined assessment of clinical parameters (salivary pH, papillary bleeding index, plaque index) and quantitative microbial load enhances the validity of the findings by integrating both host- and pathogen-related determinants. The use of standardized clinical protocols, a single calibrated examiner, and a validated PCR-based assay for pathogen quantification contributes to the reliability, reproducibility, and internal consistency of the results. Conducting the study in a real clinical setting and including a well-matched non-smoker control group further strengthens its external relevance.

Several limitations should also be considered. As a cross-sectional study, it does not permit causal inference or the evaluation of temporal changes in clinical or microbial parameters. The sample size, although sufficient for primary comparisons, limits the depth of subgroup analyses and may not fully represent the broader smoking population. The microbial panel included only selected periodontal pathogens, without capturing the full diversity of the oral microbiome or host inflammatory markers that may influence restorative outcomes. The PCR panel targeted a limited subset of pathogens and did not provide information on broader microbiome composition, which represents a methodological constraint of the study. Behavioral and environmental confounders, such as diet, oral hygiene practices, or socioeconomic status, were not systematically controlled for. Additionally, smoking intensity and cumulative exposure were not quantified in detail, which may influence the degree of clinical and microbiological alteration. These aspects should be taken into account when interpreting the findings and planning future longitudinal or mechanistic studies. In addition, the limited availability of comparable clinical studies specifically addressing the combined effects of smoking, peri-coronal microbiology, and endocrown restorations restricts direct comparison of the present findings with existing clinical evidence and highlights the need for further well-designed clinical investigations in this area.

## 5. Conclusions

This age-stratified analysis shows that smoking negatively affects the peri-coronal environment of posterior teeth restored with lithium disilicate endocrowns. Smokers exhibited lower salivary pH, higher plaque accumulation, and increased levels of orange-complex pathogens, creating conditions that may compromise adhesive marginal stability. These effects were more pronounced in older adults, suggesting a cumulative detrimental impact of long-term tobacco exposure on both periodontal and restorative parameters. Smoking status and patient age should be considered key prognostic factors when planning lithium disilicate endocrown therapy. Early risk-based screening and individualized preventive strategies may help counteract the unfavorable biological conditions identified in smokers. Longitudinal studies are needed to clarify the long-term influence of smoking-related biochemical and microbiological changes on the survival of adhesive ceramic restorations.

## Figures and Tables

**Table 1:** Salivary pH, papillary bleeding index, and dental plaque index were recorded adjacent to endocrown-restored posterior teeth, by age group, in control and study participants (mean ± standard deviation).

Age Group (Years)	Salivary pH (Control)	Salivary pH (Smokers)	PBI (Control)	PBI (Smokers)	PI (Control)	PI (Smokers)
19–25	7.05 ± 0.15	6.55 ± 0.20	0.87 ± 0.64	1.10 ± 0.25	0.83 ± 0.10	2.10 ± 0.40
26–35	6.96 ± 0.16	6.48 ± 0.20	0.77 ± 0.62	1.85 ± 0.50	0.72 ± 0.21	1.90 ± 0.35
36–45	6.88 ± 0.18	6.40 ± 0.22	0.75 ± 0.43	2.00 ± 0.50	0.80 ± 0.15	2.10 ± 0.30
46–55	6.80 ± 0.20	6.30 ± 0.25	0.82 ± 0.51	2.20 ± 0.55	0.82 ± 0.08	2.30 ± 0.35
56–65	6.70 ± 0.22	6.20 ± 0.27	1.12 ± 0.59	2.35 ± 0.60	0.70 ± 0.33	2.40 ± 0.40
66–75	6.62 ± 0.24	6.10 ± 0.28	1.25 ± 0.66	2.45 ± 0.50	0.80 ± 0.27	2.50 ± 0.38
≥76	6.55 ± 0.25	6.00 ± 0.30	1.66 ± 0.47	2.60 ± 0.48	0.86 ± 0.04	2.55 ± 0.36
Average ± SD	6.77 ± 0.21	6.29 ± 0.25	1.00 ± 0.67	2.17 ± 0.55	0.76 ± 0.24	2.27 ± 0.36

**Table 2:** Total microbial load and key periodontal pathogens quantified around endocrown-restored posterior teeth (×10^2^ CFU/mL).

Group	Total Sprouts ×10^2^	Orange Complex	Aa	Pi	Pm	Fn	En
Control (Mean ± SD)	7.80 ± 3.40	3.90 ± 2.80	0.10 ± 0.08	1.20 ± 0.60	0.15 ± 0.10	1.8 ± 0.9	0.20 ± 0.10
Study (Mean ± SD)	9.10 ± 4.20	6.80 ± 2.90	0.30 ± 0.15	3.80 ± 1.40	0.55 ± 0.30	3.6 ± 1.5	0.40 ± 0.20
*p*-value	0.04	0.01	0.02	<0.001	0.01	<0.001	0.02

Aa, *Aggregatibacter actinomycetemcomitans*; Pi, *Prevotella intermedia*; Pm, *Prevotella melaninogenica*; Fn, *Fusobacterium nucleatum*; En, *Eubacterium nodatum*. Values are expressed as ×10^2^ CFU/mL (mean ± SD). *p*-values indicate differences between the control and smoker groups.

**Table 3:** One-way ANOVA results for plaque index, papillary bleeding index, and salivary pH measured adjacent to endocrown-restored posterior teeth across age groups (total sample).

Variable	Source	Sum of Squares	df	Mean Square	F	*p*-Value
Dental plaque index * age	Between Groups	1.195	6	0.199	3.580	0.006
	Within Groups	2.393	43	0.056		
	Total	3.588	49			
Papillary bleeding index * age	Between Groups	21.572	6	3.595	12.440	0.001
	Within Groups	12.428	43	0.289		
	Total	34.000	49			
Salivary pH * age	Between Groups	7.765	6	1.294	5.705	0.001
	Within Groups	9.755	43	0.277		
	Total	17.520	49			

Note: df = degrees of freedom; F = F-statistic. The symbol ‘*’ denotes the interaction term between the clinical variable and age.

**Table 4:** One-way ANOVA results for plaque index, papillary bleeding index, and salivary pH measured adjacent to endocrown-restored posterior teeth across age groups in smokers (study group).

Variable	Source	Sum of Squares	df	Mean Squares	F	*p*-Value
Dental plaque index * age	Between Groups	1.612	6	0.269	4.228	0.002
	Within Groups	2.732	43	0.064		
	Total	4.344	49			
Papillary bleeding index * age	Between Groups	10.035	6	1.673	5.271	<0.001
	Within Groups	13.645	43	0.317		
	Total	23.680	49			
Salivary pH * age	Between Groups	3.822	6	0.637	5.171	<0.001
	Within Groups	5.298	43	0.123		
	Total	9.120	49			

Note: df = degrees of freedom; F = F-statistic. The symbol ‘*’ denotes the interaction term between the clinical variable and age.

## Data Availability

The original contributions presented in this study are included in the article. Further inquiries can be directed to the corresponding authors.

## Abbreviations

The following abbreviations are used in this manuscript:

A-PRF+	Advanced platelet-rich fibrin +
Aa	*Aggregatibacter actinomycetemcomitans*
ANOVA	Analysis of variance
APC	Article Processing Charge
CAD-CAM	Computer-aided design/Computer-aided manufacturing
CFU	Colony Forming Units
dF	Degrees of freedom
En	*Eubacterium nodatum*
Fn	*Fusobacterium nucleatum*
HSD	Honest Significant Difference
PARP-1	Poly (ADP-Ribose) Polymerase 1
PBI	Papillary bleeding index
PCR	Polymerase chain reaction
PI	Dental plaque index
Pi	*Prevotella intermedia*
Pm	*Prevotella melaninogenica*
SD	Standard deviation
Sig.	Significance
